# Prevalence of chronic musculoskeletal conditions and associated factors in Brazilian adults – National Health Survey

**DOI:** 10.1186/s12889-018-5192-4

**Published:** 2018-02-27

**Authors:** Mariana Alonso Monteiro Bezerra, Natália Hellwig, Geraldo da Rocha Castelar Pinheiro, Claudia Souza Lopes

**Affiliations:** 1 Institute of Social Medicine, Department of Epidemiology, Rio de Janeiro State University, 524 Sao Francisco Xavier St., Maracana, Block D, 7th floor, Rio de Janeiro, 20550-013 Brazil; 2 Internal Medicine Department, Rheumatology Division, Rio de Janeiro State University, Boulevard 28 de Setembro, 77 – Room 333 – 3rd floor, Rio de Janeiro, 20551-030 Brazil

**Keywords:** Brazil, Epidemiology, Cross-section study, Chronic morbidities, Musculoskeletal conditions

## Abstract

**Background:**

Chronic non-communicable diseases entail high impact on health systems in Brazil and worldwide. Among the most frequent are the musculoskeletal conditions which comprise a group of diseases that influence individuals’ physical status, quality of life and functional capacity. Epidemiological studies investigating the scale of such conditions in the adult population are scarce in Brazil. This study estimates the prevalence of chronic musculoskeletal conditions and their association with demographic, socioeconomic, behavioural and clinical factors.

**Methods:**

Cross-sectional study with data from Brazil’s 2013 National Health Survey (*Pesquisa Nacional de Saúde*), a nationwide household survey of 60,202 adults. Musculoskeletal conditions were specified by self-reported medical diagnosis of arthritis or rheumatism and self-reported spinal disorders. The variables were examined using a hierarchical model of determination. Prevalences of musculoskeletal conditions were calculated with their respective 95% confidence intervals for Brazil and its five regions. Prevalence ratios (PRs) were obtained by Poisson regression with robust variance.

**Results:**

Of the 60,202 individuals evaluated, 21.6% presented musculoskeletal conditions, with higher prevalences for females, older adults, indigenous, those living with a partner, low education, no occupational activity, those living in the South Region of Brazil, in rural areas, daily smokers, sedentary, obese, those who did not drink alcohol, with depressive symptoms or suffering from three or more chronic diseases. Multivariate analysis identified strong associations with advanced age (PR = 3.61; 95% CI 3.27-3.98), depressive symptoms (PR = 1.69; 95% CI 1.57-1.81) and multimorbidity (PR = 1.94; 95% CI 1.77-2.12).

**Conclusions:**

The results show high prevalence of musculoskeletal conditions in Brazil’s adult population. Considering the process of aging and steady growth in chronic diseases, this study underlines the need for health policies directed to prevention, treatment and rehabilitation for people affected by chronic musculoskeletal conditions.

## Background

Steady growth in chronic non-communicable diseases (CNCDs) is an important phenomenon in public health worldwide [1]. Epidemiological shifts in population and longer life expectancy leads to higher risk of CNCDs [2, 3]. This entails high costs for preventive, therapeutic and curative strategies and causes major impact on public health, exhibiting high mortality rates and multiple levels of morbidity expressed in functional impairment and poor quality of life [4–6].

Chronic musculoskeletal conditions (CMCs) jeopardise affected individuals’ quality of life and functional capacity [7–9]. Rheumatic diseases such as osteoarthritis and spinal disorders manifest pain and limitations on physical, functional and labour capacities [8]. From the socioeconomic standpoint, CMCs are among the major causes of functional disability, absence from occupational activities and early retirement, resulting in impacts on individuals and society [10–12].

In Brazil, according to a study with data from the 2003 and 2008 National Household Sample Surveys (*Pesquisa Nacional por Amostra de Domicílios*, PNAD), ‘spinal disorders’ and ‘arthritis and rheumatic diseases’ were identified as being, respectively, the second and third most prevalent causes of self-reported disease of the 12 CNCDs investigated in Brazil’s adult population [13]. Also, publications by the Community-Oriented Programme for Control of Rheumatic Diseases (COPCORD) [14] – which conducts epidemiological rheumatology studies in developing countries – estimated the prevalence of rheumatic diseases at 30.9% in the town of Montes Claros, Minas Gerais state [15], and 30.4% in a study of 578 participants in Vitória, Espirito Santo state [16]. A more recent population-based survey, also applying the COPCORD questionnaire, involved 5000 adults from the five Brazilian geographic regions. Among 1342 (26.9%) participants who presented musculoskeletal symptoms unrelated to trauma in 7 days preceding the interview, the spine was the most frequent pain site involved (76.7%) [17].

In addition to biological and physiopathological factors, there are other determinants known to be related to the presence of CMCs in the general population including the socioeconomic context, behavioural and environmental factors, such as habits and lifestyle, and clinical health condition [18–20]. Research on this subject is still very much incipient in Brazil, conducted with relatively small samples. There is a lack of epidemiological studies to provide consistent population-based estimates of CMCs, as well as its distribution and association with other determinants. Therefore, the main aim of this study was to estimate the prevalence of CMCs in Brazil’s adult population and to point demographic, socioeconomic, behavioural and clinical factors associated with these conditions.

## Methods

### Study design and sample

This cross-sectional study examines data from Brazil’s National Health Survey (*Pesquisa Nacional de Saúde*, PNS), a nationally-representative, population-based health survey comprising all five regions of Brazil, states and state capitals, with information of urban and rural areas. The main goal of the PNS was to provide data on health status and lifestyles in Brazil’s population, as regards health access, healthcare service use and healthcare funding. From August 2013 to February 2014, 69,954 households were visited and 60,202 individual adults were interviewed, resulting in an 86.1% response rate [21].

The sampling process used random and cluster sampling, divided into three stages: census tracts as primary units; households as secondary units; and an adult household member at least 18 years old as the tertiary unit, selected with equiprobability among all the adult household members, to respond to the questionnaire at time of interview and data collection. Random selection was used in those three stages. Sample weights were calculated for the primary sampling units, households and the selected member. Detailed information on the sampling and data collection have been published previously [21, 22]. All PNS data are available on the Brazilian Institute of Geography and Statistics (*Instituto Brasileiro de Geografia e Estatística*, IBGE) website at: http://www.ibge.gov.br/home/estatistica/populacao/pns/2013/default_microdados.shtm.

The PNS was approved by Brazil’s National Research Ethics Committee (*Comissão Nacional de Ética em Pesquisa*, CONEP) with the National Health Council (*Conselho Nacional de Saúde*) Resolution No. 466/12 (No. 328159, June 26th, 2013), and all participants signed a free and informed consent at interview.

### Measurements

In this study, the variable of interest was specified as the presence of CMCs, obtained by self-reported physician diagnosis of arthritis or rheumatism and self-reported spinal problems, chronic back or neck pain, lower back pain, sciatic pain or problems in vertebral spine or intervertebral discs.

The demographic variables were stratified by sex (“male” and “female”), age group (“18-29”, “30-39”, “40-49”, “50-59”, “60 or more” years) and self-reported skin colour or race (“white”, “black”, “mixed”, “yellow” or “indigenous”). The socioeconomic variables included marital status (“living with partner” or “living without partner”), schooling level (“illiterate or elementary education incomplete”, “elementary education completed or high school education incomplete”, “high school completed or college incomplete”, “higher education complete”), occupational status (“yes” or “no”), geographical area of residence (rural or urban) and region of residence (“North”, “Northeast”, “Midwest”, “Southeast” and “South”).

The behavioural variables were current smoking, alcohol consumption, and weekly leisure time physical activity. Current smoking was evaluated by the question: “Do you currently smoke any tobacco product?”, with three responses categories: “Yes, daily”, “Yes, less than daily” and “No, I do not currently smoke”. Alcohol consumption was first defined by the question: “How often do you usually drink an alcoholic beverage?” and responses were categorized as “Never” and “Less than once a month or more”. In order to characterise differences between sexes in moderate or abusive alcohol consumption, two separate questions were asked for men and women: male participants were asked “In the past 30 days, did you drink five or more doses of alcoholic beverage on any single occasion?”, while female participants were asked “In the past 30 days, did you drink four our more doses of alcoholic beverage on a single occasion?”, with “no” or “yes” as the possible responses. Then, a new variable was created for consumption of alcohol, with the following categories of responses: “Never drinks”, “Drinks less than once a month or not in excess” and “Drinks in excess”. Weekly leisure physical activity was considered for those who reported some leisure physical activity in the last week and duration of the activity, being then categorized as: “more than 150 min”, “less than 150 min” and “physically inactive”).

For the clinical variables, the anthropometric biomarker used was Body Mass Index (BMI) in four categories: underweight (less than 18.5 Kg/m^2^), eutrophic (18.5-24.9 Kg/m^2^), overweight (25.0-29.9 Kg/m^2^) and obese (more than 30.0 Kg/m^2^), using self-reported weight and height information at interview. The translated and validated Brazilian version of the Patient Health Questionnaire-9 (PHQ-9) was used to measure prevalence of depression, with cut-off at 10 points or more [23]. A multimorbidity score (“none”, “one”, “two”, “three or more”) was created from self-reported medical diagnosis of the following CNCDs: arterial hypertension, diabetes, hypercholesterolemia, asthma, cardiovascular diseases, stroke, chronic obstructive pulmonary disease, chronic kidney failure, cancer, mental disorders and other chronic diseases.

### Statistical analysis

Data analysis were performed using Stata 12 statistical software. The appropriate weights deriving from the complex sampling design were taken into account in all the analyses by using the *survey* command with the prefix *svy*. Descriptive analyses for Brazil and its five regions were performed for all the study variables, with relative frequencies of prevalence of CMCs and respective 95% confidence intervals (95% CI). For the associated factors, bivariate and multivariate analyses were conducted using Poisson regression with robust variance, providing measures of association as crude and adjusted prevalence ratios (PR) and their respective 95% CIs [24].

The adjusted analysis used a hierarchical model of determination [25]. This type of analysis considers the effect of each variable to the outcome, controlling for possible confounder effects between proximal and distal variables. The individual characteristics (sex, age and self-reported skin colour or race) were considered to be distal variables that could act as determinants of demographic and socioeconomic status (marital status, schooling level, occupational status, geographical area of residence, region of residence) which in turn could interfere in lifestyle and behavioural variables (smoking, drinking and level of physical activity). Lastly, clinical variables, such as anthropometric features (BMI) and other chronic diseases (depression and multimorbidity) could lead to CMCs.

## Results

Descriptive characteristics of the sample and estimated adult population are shown in Table 1. Prevalence of CMCs in Brazil’s adult population was estimated at 21.6%. The South region showed the highest prevalence, estimated at 26.5% (95% CI 24.8-28.3), followed by 22.0% (95% CI 20.8-23.2) in the Northeast, 21.0% (95% CI 19.3-22.6) in the North, 20.2% (95% CI 18.8-21.2) in the Midwest, and 20.1% (95% CI 19.1-21.2) in the Southeast region.

**Table 1:** Characteristics and prevalence of chronic musculoskeletal conditions in the Brazilian estimated population, 2013

Variable	Sample	Estimated Population		Musculoskeletal conditions	
	*n*	*N*	%	Yes % (95% CI)	No % (95% CI)
Sex					
Male	25.920	68.916.470	47.1	17.6 (16.7-18.4)	82.4 (81.6-83.2)
Female	34.282	77.391.988	52.9	25.3 (24.4-26.1)	74.7 (73.9-75.6)
Age					
18-29 years	14.321	38.157.850	26.1	9.7 (8.8-10.7)	90.2 (89.3-91.1)
30-39 years	14.269	31.643.091	21.6	15.5 (14.5-16.6)	84.4 (83.4-85.5)
40-49 years	11.405	26.423.124	18.1	23.7 (22.3-25.1)	76.3 (74.9-77.7)
50-59 years	9.030	23.676.562	16.2	30.9 (29.2-32.5)	69.1 (67.4-70.8)
60 years or more	11.177	26.407.831	18.0	35.8 (34.2-37.4)	64.2 (62.6-65.8)
Skin colour					
White	24.106	69.443.157	47.5	22.6 (21.6-23.5)	77.4 (76.5-78.3)
Black	5.631	13.454.442	9.2	21.0 (19.0-23.0)	79.0 (77.0-80.9)
Yellow	533	1.371.897	0.9	16.7 (11.4-21.9)	83.3 (78.0-88.6)
Mixed race	29.512	61.419.933	42.0	20.8 (19.9-21.7)	79.2 (78.3-80.1)
Indigenous	417	619.030	0.4	29.3 (21.3-37.3)	70.7 (62.7-78.7)
Marital status					
With partner	34.522	89.537.328	61.2	23.6 (22.7-24.4)	76.4 (75.5-77.2)
Without partner	25.680	56.771.130	38.8	18.6 (17.8-19.3)	81.4 (80.6-82.2)
Schooling level					
Illiterate/Elementary education incomplete	24.083	57.041.784	39.0	28.7 (27.6-29.8)	71.3 (70.2-72.4)
Elementary completed/High school incomplete	9.215	22.761.619	15.5	19.1 (17.6-20.5)	80.9 (79.5-82.4)
High school completed/College incomplete	19.149	48.109.933	32.9	16.2 (15.3-17.1)	83.8 (82.9-84.7)
Higher education complete	7.603	18.395.122	12.6	17.4 (15.8-19.0)	82.6 (81.0-84.2)
Geographical area					
Rural	10.957	20.176.036	13.8	24.5 (22.7-26.2)	75.5 (73.7-77.3)
Urban	49.245	126.132.422	86.2	21.2 (20.5-21.9)	78.8 (78.1-79.5)
Occupational status^a^					
Yes	34.102	87.164.817	59.6	18.2 (17.4-19.0)	81.8 (81.0-82.6)
No	24.443	59.143.641	40.4	26.4 (25.4-27.4)	73.6 (72.6-74.5)
Smoking					
Yes, daily	7.334	18.628.350	12.7	25.6 (23.9-27.3)	74.4 (72.7-76.1)
Yes, less than daily	1.395	2.891.025	2.0	18.4 (14.9-21.9)	81.6 (78.1-85.1)
No	51.473	124.789.083	85.3	21.1 (20.4-21.8)	78.9 (78.2-79.6)
Drinking per month					
Excessive	8.104	19.972.635	13.6	16.6 (15.2-18.0)	83.4 (82.0-84.8)
Moderate	14.898	39.152.545	26.8	20.5 (19.3-21.6)	79.5 (78.3-80.7)
None	37.200	87.183.278	59.6	23.3 (22.5-24.1)	76.7 (75.8-77.5)
Leisure physical activity					
More than 150 min weekly	11.212	28.250.116	19.3	17.3 (16.0-18.6)	82.7 (81.4-83.9)
Less than 150 min weekly	6.137	16.339.629	11.2	21.0 (19.2-22.8)	79.0 (77.2-80.8)
Inactive	42.853	101.718.713	69.5	22.9 (22.2-23.7)	77.1 (76.3-77.8)
BMI					
Underweight	1.705	7.031.280	6.7	18.7 (13.8-23.6)	81.3 (76.4-86.1)
Eutrophic	15.412	54.132.226	37.1	19.1 (18.1-20.2)	80.9 (79.8-81.9)
Overweight	14.969	58.011.497	41.4	22.6 (21.4-23.8)	77.4 (76.1-78.6)
Obese	6.284	27.060.415	14.8	23.0 (22.1-23.9)	77.0 (76.1-77.9)
Depression					
Yes	5.051	11.553.035	7.9	45.1 (42.8-47.4)	54.9 (52.6-57.2)
No	55.151	134.755.423	92.1	19.6 (19.0-20.3)	80.4 (79.7-81.0)
Multimorbidity					
None	29.477	85.955.634	58.8	14.4 (13.7-15.2)	85.6 (84.8-86.3)
One	12.245	35.946.588	24.6	26.6 (25.2-27.9)	73.4 (72.1-74.8)
Two	5.416	15.692.311	10.7	37.3 (34.9-39.7)	62.7 (60.3-65.1)
Three or more	3.028	8.682.057	5.9	49.4 (46.5-52.3)	50.6 (47.7-53.4)

*Legend*: *n* Sample, *N* Estimated population, ^a^variable with missing data *n* = 58,545, *BMI* Body Mass Index

Table 2 shows prevalence of CMCs by region of Brazil and by demographic, socioeconomic and lifestyle factors, BMI and clinical conditions. CMCs were more prevalent among women (25.3%), increased with advancing age (36% more frequent in the elderly) and were also greater in self-reported indigenous participants (nearly 30%). The highest prevalence of CMCs was found in individuals living with partners (23.6%), individuals with lower schooling level (28.7%), those who were not working (26.4%) and those living in rural areas (24.5%). In relation to lifestyle habits, prevalence of CMCs was estimated at 44% in smokers. Individuals with no alcohol consumption showed higher prevalences of CMCs (23.3%). Prevalence of CMCs was higher in physically inactive individuals (23.0%) than in those who engaged in physical activity for more than 150 min per week (17.3%). In addition, prevalence of CMCs was greater in overweight (22.6%) and obese individuals (23.0%). Prevalence of CMCs increased with number of self-reported diseases as multimorbidities, affecting 26.6% of individuals with one chronic disease, 37.3% of those with two and 49.4% of those with three or more morbidities, as well as CMCs were prevalent in approximately 45% of the depressed individuals.

**Table 2:** Prevalence of chronic musculoskeletal conditions by regions of Brazil, 2013

Variable	North	Northeast	Midwest	Southeast	South
	% (95% CI)	% (95% CI)	% (95% CI)	% (95% CI)	% (95% CI)
Sex					
Male	18.1 (16.1-20.2)	19.0 (17.5-20.4)	14.5 (12.9-16.1)	15.7 (14.3-17.0)	21.7 (19.5-24.1)
Female	23.6 (21.5-25.7)	24.6 (23.1-26.2)	25.6 (23.7-27.2)	24.1 (22.6-25.5)	30.8 (28.5-33.2)
Age					
18-29 years	11.9 (9.9-13.8)	10.2 (8.8-11.6)	8.6 (6.7-10.6)	7.8 (6.3-9.3)	13.6 (10.2-17.0)
30-39 years	18.1 (15.7-20.4)	17.7 (15.7-19.6)	15.1 (12.8-17.4)	13.6 (11.8-15.4)	16.2 (13.6-18.7)
40-49 years	23.3 (19.7-26.8)	24.5 (22.1-26.9)	21.6 (19.0-24.3)	22.3 (19.9-24.8)	27.4 (23.8-31.0)
50-59 years	33.7 (28.5-38.9)	31.6 (28.8-34.4)	30.2 (26.6-33.8)	27.4 (24.7-30.2)	39.0 (34.6-43.3)
60 years or more	34.0 (29.4-38.5)	36.1 (33.4-38.9)	35.7 (32.3-39.1)	33.7 (31.0-36.3)	42.8 (39.1-46.6)
Skin colour					
White	21.9 (18.9-24.8)	21.8 (20.1-23.5)	21.6 (19.8-23.5)	21.3 (19.8-22.8)	26.1 (24.1-28.0)
Black	24.0 (19.0-28.9)	23.8 (20.2-27.4)	16.0 (12.1-19.8)	18.5 (15.5-21.6)	24.9 (18.7-31.2)
Yellow	18.3 (4.6-31.9)	15.0 (8.4-21.6)	17.2 (7.5-27.0)	16.7 (8.2-25.1)	19.6 (−0.1-39.4)
Mixed race	20.3 (18.4-22.2)	21.7 (20.3-23.1)	19.7 (17.8-21.6)	18.7 (16.9-20.5)	29.3 (25.2-33.4)
Indigenous	22.1 (8.7-35.5)	29.8 (16.4-43.3)	20.2 (−3.2-43.6)	33.6 (16.4-50.7)	40.6 (8.7-72.5)
Marital status					
With partner	21.6 (19.7-23.5)	23.4 (21.8-24.9)	21.4 (19.7-23.2)	23.0 (21.5-24.5)	27.4 (25.2-29.6)
Without partner	19.9 (17.6-22.1)	19.8 (18.3-21.3)	18.1 (16.4-19.9)	16.0 (14.7-17.3)	24.9 (22.5-27.3)
Schooling level					
Illiterate/Elementary education incomplete	26.7 (24.1-29.3)	27.7 (26.0-29.3)	26.4 (24.3-28.6)	27.2 (25.1-29.4)	36.9 (34.1-39.7)
Elementary completed/High school incomplete	18.0 (15.0-20.9)	18.3 (15.3-21.2)	18.8 (15.5-22.0)	17.9 (15.4-20.4)	24.2 (20.6-27.9)
High school completed/College incomplete	16.2 (14.1-18.3)	15.9 (14.4-17.4)	15.1 (13.1-17.1)	15.7 (14.2-17.2)	18.7 (16.1-21.4)
Higher education complete	17.3 (13.2-21.4)	17.0 (14.5-19.5)	17.5 (14.7-20.4)	17.2 (14.6-19.8)	18.2 (14.5-21.9)
Geographical area					
Rural	21.1 (17.4-24.7)	23.6 (20.9-26.2)	19.3 (15.9-22.7)	23.4 (18.5-28.3)	32.6 (29.1-36.1)
Urban	20.9 (19.1-22.8)	21.5 (20.2-22.8)	20.3 (18.8-21.7)	19.9 (18.8-21.0)	25.5 (23.5-27.5)
Occupational status *					
Yes	19.5 (17.4-21.6)	19.4 (17.8-21.0)	15.8 (14.2-17.4)	16.4 (15.1-17.8)	21.7 (19.5-23.8)
No	23.1 (20.9-25.4)	24.5 (23.0-26.1)	27.7 (25.4-30.0)	25.5 (23.8-27.2)	35.4 (32.5-38.2)
Smoking					
Yes, daily	27.6 (22.9-32.2)	26.3 (23.6-29.0)	20.6 (17.0-24.2)	24.6 (21.7-27.5)	28.9 (24.5-33.3)
Yes, less than daily	21.1 (14.3-27.8)	16.8 (11.9-21.7)	14.8 (8.3-21.3)	17.5 (9.9-25.0)	23.9 (13.7-34.0)
No	20.2 (18.6-21.9)	21.5 (20.3-22.8)	20.2 (18.8-21.6)	19.5 (18.3-20.7)	26.2 (24.2-28.2)
Drinking per month					
Excessive	17.9 (14.2-21.5)	16.2 (14.2-18.2)	15.3 (12.4-18.2)	15.4 (12.9-17.8)	21.7 (16.5-26.9)
Moderate	21.3 (18.1-24.5)	19.6 (17.6-21.6)	18.0 (15.6-20.4)	19.6 (17.6-21.5)	24.2 (21.3-27.0)
None	21.5 (19.6-23.4)	24.4 (22.9-25.9)	22.4 (20.7-24.1)	21.4 (20.0-22.8)	29.2 (26.9-31.4)
Leisure physical activity					
More than 150 min weekly	14.8 (12.4-17.2)	18.2 (16.1-20.4)	15.7 (13.5-17.9)	16.5 (14.2-18.7)	20.5 (17.3-23.7)
Less than 150 min weekly	18.6 (14.5-22.6)	23.3 (19.7-26.9)	17.2 (14.2-20.2)	18.4 (15.2-21.5)	25.5 (21.3-29.6)
Inactive	22.9 (20.8-24.9)	22.8 (21.6-24.0)	22.1 (20.5-23.7)	21.4 (20.1-22.7)	28.5 (26.4-30.5)
BMI					
Underweight	17.8 (11.1-24.6)	16.5 (10.1-23.0)	11.6 (5.6-17.7)	20.6 (10.2-30.6)	22.2 (10.3-34.0)
Eutrophic	18.3 (15.9-20.7)	20.4 (18.3-22.4)	16.7 (14.9-18.6)	17.7 (16.0-19.4)	22.8 (20.2-25.5)
Overweight	21.9 (19.1-24.8)	23.5 (21.4-25.6)	20.5 (18.1-22.8)	21.8 (19.8-23.9)	25.0 (22.1-27.9)
Obese	22.1 (19.8-24.3)	22.4 (21.0-23.8)	23.4 (21.1-25.6)	20.9 (19.3-22.6)	31.2 (28.5-34.0)
Depression					
Yes	43.4 (37.8-49.0)	45.7 (42.0-49.3)	41.6 (36.3-46.9)	42.3 (38.0-46.5)	53.3 (48.2-58.3)
No	19.5 (17.8-21.2)	19.9 (18.7-21.1)	18.2 (16.9-19.6)	18.3 (17.2-19.4)	23.9 (22.1-25.6)
Multimorbidity					
None	16.7 (14.6-18.8)	15.7 (14.4-17.1)	13.8 (12.2-15.4)	12.1 (10.9-13.2)	18.1 (15.7-20.4)
One	26.9 (23.1-30.7)	27.6 (25.1-30.1)	26.0 (22.7-29.3)	24.2 (21.9-26.5)	31.5 (28.4-34.7)
Two	37.2 (31.9-42.4)	37.5 (33.4-41.7)	39.3 (34.0-44.6)	36.4 (32.2-40.6)	38.5 (33.3-43.6)
Three or more	57.7 (47.4-67.9)	48.6 (43.4-53.8)	41.4 (35.1-47.7)	47.5 (42.8-52.0)	57.4 (51.0-63.7)

*Legend*: *BMI* Body Mass Index

*Variable with missing data *n* = 58,545

Table 3 shows the measures of association between the independent variables and the presence of CMCs. Crude analysis show all variables were associated with CMCs. Adjusted analysis revealed that being a woman increased prevalence of CMCs by 40% (PR = 1.40; 95% CI 1.32-1.47). Prevalence of CMCs increases with age and in the elderly it is nearly 260% higher than in young adults (PR = 3.61; 95% CI 3.27-3.98). Also, self-reported yellow individuals showed lesser CMCs (PR = 0.74; 95% CI 0.55-0.99), while prevalence in self-reported indigenous individuals was 37% greater, both compared to white individuals.

**Table 3:** Associated factors with presence of musculoskeletal conditions in the estimated population of Brazilian adults, 2013

Variable	Crude PR (95% CI)	Adjusted PR (95% CI)
Sex		
Male	1.00	1.00
Female	1.44 (1.36-1.52)	1.40 (1.32-1.47)
Age		
18-29 years	1.00	1.00
30-39 years	1.59 (1.43-1.78)	1.58 (1.42-1.77)
40-49 years	2.43 (2.18-2.70)	2.40 (2.16-2.66)
50-59 years	3.16 (2.85-3.51)	3.15 (2.84-3.49)
60 years or more	3.67 (3.33-4.05)	3.61 (3.27-3.98)
Skin colour		
White	1.00	1.00
Black	0.93 (0.84-1.03)	0.95 (0.86-1.04)
Yellow	0.74 (0.54-1.02)	0.74 (0.55-0.99)
Mixed race	0.92 (0.87-0.97)	1.00 (0.95-1.06)
Indigenous	1.30 (0.98-1.72)	1.37 (1.05-1.79)
Marital status		
With partner	1.27 (1.20-1.34)	1.17 (1.11-1.24)
Without partner	1.00	1.00
Schooling level		
Illiterate/Elementary education incomplete	1.65 (1.50-1.82)	1.37 (1.24-1.51)
Elementary completed/High school education incomplete	1.10 (0.98-1.23)	1.24 (1.10-1.40)
High school education completed/College incomplete	0.93 (0.84-1.03)	1.12 (1.00-1.24)
Higher education complete	1.00	1.00
Geographical area		
Rural	1.15 (1.08-1.23)	1.03 (0.96-1.10)
Urban	1.00	1.00
Region		
North	1.04 (0.95-1.13)	1.16 (1.07-1.26)
Northeast	1.09 (1.02-1.17)	1.10 (1.03-1.18)
Midwest	1.00 (0.92-1.08)	1.06 (0.98-1.14)
Southeast	1.00	1.00
South	1.31 (1.22-1.42)	1.28 (1.19-1.39)
Occupational status		
Yes	1.00	1.00
No	1.46 (1.38-1.54)	1.02 (0.96-1.09)
Smoking		
Yes. daily	1.21 (1.12-1.30)	1.13 (1.05-1.22)
Yes. less than daily	0.87 (0.72-1.05)	0.96 (0.80-1.16)
No	1.00	1.00
Drinking/month		
Excessive	0.71 (0.64-0.78)	1.07 (0.98-1.18)
Moderate	0.88 (0.82-0.93)	1.09 (1.02-1.17)
None	1.00	1.00
Leisure physical activity		
More than 150 min weekly	0.75 (0.69-0.81)	0.95 (0.88-1.02)
Less than 150 min weekly	0.91 (0.83-1.00)	1.15 (1.05-1.25)
Inactive	1.00	1.00
BMI		
Underweight	0.98 (0.76-1.26)	0.98 (0.77-1.25)
Eutrophic	1.00	1.00
Overweight	1.18 (1.10-1.28)	1.06 (0.99-1.14)
Obese	1.20 (1.12-1.28)	0.95 (0.89-1.01)
Depression		
Yes	2.29 (2.16-2.44)	1.69 (1.57-1.81)
No	1.00	1.00
Multimorbidity		
None	1.00	1.00
One	1.83 (1.71-1.96)	1.42 (1.32-1.53)
Two	2.53 (2.34-2.73)	1.69 (1.55-1.84)
Three or more	3.35 (3.10-3.63)	1.94 (1.77-2.12)

*Legend*: *PR* Prevalence ratio

Prevalence of CMCs was observed to be 17% greater in individuals who lived with a partner. Using the Southeast region as reference, CMCs were 28% more prevalent in the South region, followed by the North (PR = 1.16; 95% CI 1.07-1.26) and Northeast (PR = 1.10; 95% CI 1.03-1.18), while there was no statistically significant difference between the Midwest and Southeast regions. There was an inverse relation between individuals’ level of schooling and prevalence of CMCs, which was nearly 40% greater in those with no schooling or with incomplete elementary education as compared with participants who had completed higher education (PR = 1.37; 95% CI 1.24-1.51) and 24% greater in those with incomplete high school education. No statistically significant association were found between CMCs and both work activity and area of residence.

The adjusted analysis showed 13% greater prevalence of CMCs in daily smokers compared with individuals who never smoked. There was a discrete association between CMCs and moderate drinking as compared with individuals who drank no alcohol. The protective effect of physical activity was not present in the adjusted model, although it should be noted that PR for CMCs was greater in those who engaged in physical exercise for less than 150 min per week compared with inactive individuals (PR = 1.14; 95% CI 1.05-1.25). In this study, no statistically significant association was found for BMI categories.

Depression increased prevalence of CMCs by approximately 70%. Prevalence of CMCs was higher in individuals with multimorbidity and increased significantly with the adding number of simultaneous morbidities. The strongest association was found in individuals with three or more self-reported morbidities (PR = 1.94; 95% CI 1.77-2.12).

## Discussion

This study pointed population-based prevalence of self-reported CMCs and associated factors in Brazil’s adult population, aggregating rheumatic diseases and spinal disorders, using data from the country’s first major nationally-representative population health survey. Prevalence of CMCs in Brazil can be considered high, given that they are reported by one in five adults. This finding agrees with other estimates in the literature. Data from the 2008 PNAD show that approximately 19% of self-reported chronic diseases were rheumatic musculoskeletal diseases and spinal problems [13]. Prior studies have pointed a 30.9% prevalence of musculoskeletal diseases in Minas Gerais state [15], while other study with a sample of 5000 individuals in 16 of Brazil’s state capitals estimated the prevalence of CMCs unrelated to trauma at 12.8% [17]. The differences in the estimated prevalence of these studies may be explained by the methodologies applied, the musculoskeletal conditions definitions, the measurement instruments used and the population sample.

In this study, as in the literature cited, women displayed 40% greater prevalence of CMCs than men. This finding may be explained, at least in part, by women’s being more inclined to report health problems in population surveys, as well as being more frequent users of health services [26]. As expected, a strong and increasing association was observed between age and CMCs. Given longer life expectancy, the relation between age and increasing prevalence of chronic diseases and functional disability demands greater attention from health policymakers with a view to adjusting management of these conditions in the population. Prior studies using PNAD data have found greater prevalence of CNCDs in indigenous people [13]. The CMCs evaluated in this study were also more prevalent in the indigenous population, although it should be noted that the sample contained only a small number of self-reported yellow and indigenous individuals and, for that matter, these prevalences may have been overestimated.

CMCs were more prevalent in participants living with a partner than in those living alone, although studies have shown beneficial effects of living with a life partner during chronic disease [27–29]. At population level, lower education is associated with higher morbidity, disability and less health care [30]. In Brazil, despite its public, free and universal national health system, the cost of care for a chronic disease is still extremely high, contributing to family financial insufficiency in a kind of vicious circle in which expenses have to be met by cutting spending on other essentials, such as healthy food, education and well-being [31]. Greater prevalence of CMCs was found among those living in the South region of Brazil, and this finding may be justified by greater health service use in the South of Brazil. The PNS also found high prevalences of self-reported CNCDs in this region [22], as did a trend study of health inequality and prevalence of CNCDs in Brazil using 2003 to 2008 PNAD data [13]. This finding contrasts with the results of a COPCORD study of prevalence in Brazil, which found prevalence of musculoskeletal symptoms was highest in the North region and lowest in the South [17]. This inconsistency in the study findings may possibly result from sampling and methodological differences between the studies, given that this study used self-reported measures.

Studies indicate that the association with currently smoking, and partly with formerly smoking, can be explained by the pharmacological effect of tobacco smoke, which interferes biologically in the processing of pain-related thresholds and also because tobacco acts as a potential pro-inflammatory agent, causing alterations in the nutrition and healing of musculoskeletal and peripheral tissues [32–34]. This study found only slightly greater prevalence of CMCs (13%) in those who smoked daily than in non-smokers. The literature is still inconsistent over the association between alcohol drinking and musculoskeletal diseases. This study found a borderline PR for moderate drinking. One systematic review found a similar result for moderate drinking and chronic lower back pain [35].

Depression and multimorbidity were strongly associated with CMCs. This association could be explained as depression and multiple concomitant chronic diseases exert an influence on the psychosomatization manifesting as physical disorders, including CMCs, and may lead to aggravation of immunological and physical symptoms [36, 37]. The relationship between symptom amplification and CMCs has been described previously for other chronic diseases, such as asthmatics, diabetics, burn victims and chronic pain, as well as in functional disability, while psychosomatization also interferes in self-control and health perception mechanisms, increasing adverse issues related to prognosis, excessive consumption of health care and more physiopathological mechanisms of chronic diseases [38]. It is clearly difficult to establish causality between depression and CMCs, because there is a complex bidirectional relationship, a model of reciprocal influence, in which mental disorders can lead to organic diseases and these CMCs, in turn, can lead to mental disorders [39]. Even though an association was found in this study, the reciprocity between depression and CMCs makes it difficult to determine the temporal relationship between these events, particularly because of the cross-sectional design of this study.

The findings of this study largely agree with the determinants associated factors with CNCDs as most consistently described in the scientific literature. In Brazil, Barros et al. also demonstrated associations of having at least one CNCD with advancing age, female sex, indigenous race, lower education, and in individuals with health insurance, migrants from other states, urban dwellers and in residents of the South region [13].

Attention should be drawn to the limitations of this study. This study could not characterise CMCs according to bodily sites and structures, because the 2013 PNS included no such questions. Another possible limitation was response bias regarding the definition of chronic musculoskeletal conditions, because the evaluation of arthritis and spinal problems in the questionnaire was based on self-reported physician diagnoses. However, according to the literatures, prevalence of physical morbidity through this type of question has been considered as being valid and reliable in epidemiological studies. [40]. In addition, there can be no disregarding to the possibility of the reverse causality bias inherent to cross-sectional studies, which restricts the associations findings justified by the absence of temporality and absence of follow up for occurrences of the event of interest, making it hard to demonstrate the cause and effect relationship between levels of exposure and CMCs as the outcome.

One of the main advantages of this study rely on the fact that the PNS assured a population-based household survey with epidemiological reliable data and low rates of loss and refusal. The PNS was the first major health survey conducted in Brazil so far and its maintenance in future will be imperative to develop and evaluate trends studies about CNCDs, and particularly CMCs. It is hoped that further cross-sectional studies will be able to add information on the analysis of therapeutic healthcare services used by this population in Brazil. In addition, longitudinal studies could be conducted to ascertain estimates of the incidence and persistence of these CMCs in Brazil’s population.

## Conclusions

This study found high prevalence of self-reported CMCs characterised as spinal problems and rheumatic diseases, which affected about one fifth of Brazil’s adult population. It contributes with substantial findings for health systems in Brazil, primarily for public health, calls for thinking and underlines the impact of CMCs and their associated factors, which include advanced age, multimorbidity and depression, attesting to the need for health policies, investments in human resources in health and services related to prevention, treatment and rehabilitation for those individuals with CMCs, which are still increasing considerably in Brazil and worldwide.

## Abbreviations

BMI: Body Mass Index
CI: Confidence Interval
CMC: Chronic Musculoskeletal Condition
CNCD: Chronic Non-Communicable Disease
CONEP: National Research Ethics Committee (*Comissão Nacional de Ética em Pesquisa*)
COPCORD: Community-Oriented Programme for Control of Rheumatic Diseases
IBGE: Brazilian Institute of Geography and Statistics (*Instituto Brasileiro de Geografia e Estatística*)
PHQ-9: Patient Health Questionnaire-9
PNAD: National Household Sample Survey (*Pesquisa Nacional por Amostra de Domicílios*)
PNS: National Health Survey (*Pesquisa Nacional de Saúde*)
PR: Prevalence Ratio
